# Analysis of different techniques for injection of the interspinal space in horses

**DOI:** 10.1111/evj.14515

**Published:** 2025-05-09

**Authors:** Dorothea Tress, Simon Hennessy, Roswitha Merle, Katharina Charlotte Jensen, Christoph Lischer, Anna Ehrle

**Affiliations:** ^1^ Equine Clinic, Veterinary Hospital School of Veterinary Medicine, Freie Universität Berlin Berlin Germany; ^2^ Anglesey Lodge Equine Hospital Kildare Ireland; ^3^ Institute of Veterinary Epidemiology and Biostatistics School for Veterinary Medicine, Freie Universität Berlin Berlin Germany

**Keywords:** back, computed tomography, horse, injection, interspinous ligament, spinous processes

## Abstract

**Background:**

Impingement of spinous processes (SPs) is commonly diagnosed in the equine athlete. For diagnostic and therapeutic purposes, local injections are performed at the level of the space between adjacent spinous processes in affected horses.

**Objectives:**

To assess the accuracy of different techniques for the local injection of the interspinal space in the equine thoracolumbar spine.

**Study Design:**

Ex vivo experimental study.

**Methods:**

Equine thoracolumbar spine specimens were used to compare three techniques for needle insertion (midline; bilateral abaxial; unilateral oblique), two needles (20G—1½″; 20G—3½″) and two volumes (5 mL; 20 mL) for local injection of the interspinal space. Additionally, needle insertion based on radiographic, ultrasonographic guidance, or palpation was assessed. Computed tomographic analysis and anatomical dissection were performed to evaluate the distribution of the injected volume.

**Results:**

The most accurate injection of the interspinous ligament was achieved when the midline injection method using a 20G—1½″ short needle and a volume of 5 mL was used. Wide distribution of the injected volume was observed when the bilateral abaxial injection technique was utilised. The unilateral oblique injection technique led to significantly asymmetrical unilateral distribution of the injectate. Radiographic guidance did not increase the accuracy of the injection.

**Conclusion:**

The midline injection method is the most reliable technique for the targeted injection of the thoracolumbar interspinal space.

## 1 INTRODUCTION

Impingement of spinous processes (SPs) is a common radiographic finding in the equine thoracolumbar vertebral column that can be associated with back pain and result in poor performance in the equine athlete.^1^, ^2^ The condition is defined as narrowing of the space between two spinous processes (SPs), also commonly named dorsal spinous processes (DSPs), to <4 mm.^3^, ^4^, ^5^

Local injection of the interspinal space at the level of the SP summits is common practice for the accurate diagnosis and treatment in horses with back pain.^6^, ^7^ Several approaches are described for the injection of the equine thoracolumbar vertebral column region.^6^, ^8^, ^9^ The results of recent surveys confirmed that equine orthopaedic specialists mainly utilise three different injection techniques with significant variation concerning the needles and volumes used for local injection of the thoracolumbar interspinal space.^7^, ^10^ Whilst the injection of the thoracolumbar articular process joints^8^, ^11^ and the sacroiliac joints^12^, ^13^, ^14^ have been investigated, the evaluation and detailed comparison of the different techniques available for the local injection used in horses with impinging SPs is lacking.

The aim of this study was to identify the most reliable technique for injection of the interspinous ligament (ISL) in the interspinal space of the equine thoracolumbar vertebral column. Based on computed tomographic imaging and anatomical dissection, three different techniques for injection as well as two needle sizes, two volumes, and imaging techniques used to guide the injection were assessed in equine vertebral column specimens free of severe SP pathology.

It was hypothesised that placing one needle across the interspinal space in an oblique direction would be most reliable. It was additionally hypothesised that the use of a 20G—1½″ short needle instead of a 20G—3½″ spinal needle would reduce the likelihood of inadvertent injection of the thoracolumbar articular process joints and that a volume of 5 mL would result in less diffusion toward the epaxial musculature when compared with a larger volume of 20 mL. It was anticipated that radiographic confirmation is not required and that ultrasonographic guidance would be sufficient for accurate needle positioning.

## 2 MATERIALS AND METHODS

### 2.1 Animals and samples

Based on a previously described protocol, a sample size of 12 thoracolumbar vertebral column specimens was initially collected from horses euthanised for reasons unrelated to a history of spinal pathology.^15^ The following breeds with an average age of 17 years (8–28 years) and a weight range of 500–680 kg were represented: Warmblood (*n* = 10); Polo horse (*n* = 1) and Arabian horse (*n* = 1). Specimens were excised between the eighth thoracic (T8) and the sixth lumbar (L6) vertebrae, with the ribs transected at the level of the costal angle and all soft tissues including the epaxial musculature and skin left intact. Before any injection, radiographic examination of the thoracolumbar vertebrae T8–L6 (latero‐lateral projection; Gierth HF 1000, 84 kV, 20 mA direct digital radiography) was performed. Clip‐marks (small area where the hair was clipped) were subsequently prepared where circular, radiopaque markers (8 mm diameter) had been attached along the dorsal aspect of T12, T16, and L3 during the initial radiographic examination. Impingement of the SP was documented and graded as mild, moderate, or severe. Specimens with mild to moderate SP pathological findings described as lesion grade 1–3 by Zimmermann et al. (2011)^5^ were included, and specimens with severe findings at the level T11/12, T14/15, T17/18, L2/3 described as SP lesion grade 4–7^5^ were excluded from the study.

### 2.2 Experimental protocol

**FIGURE 1:**
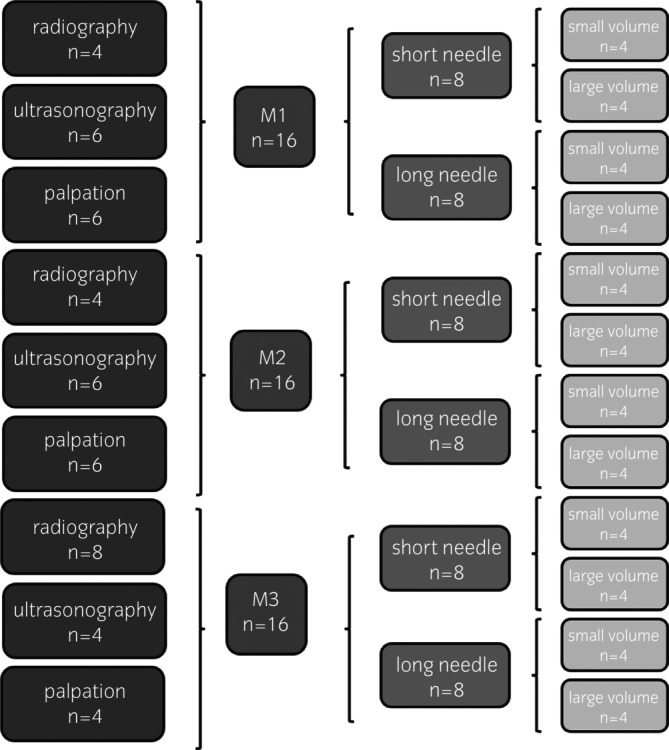
Flow chart illustrating system for injection of equine spinal specimens with selected method, needle, volume and guidance for injection (M1 = insertion of one needle in midline; M2 = insertion of two needles bilateral abaxial, left and right to the interspinal space (leading to a total number of 32 needles inserted for this method); M3 = insertion of one needle in an oblique direction = unilateral oblique).

**FIGURE 2:**
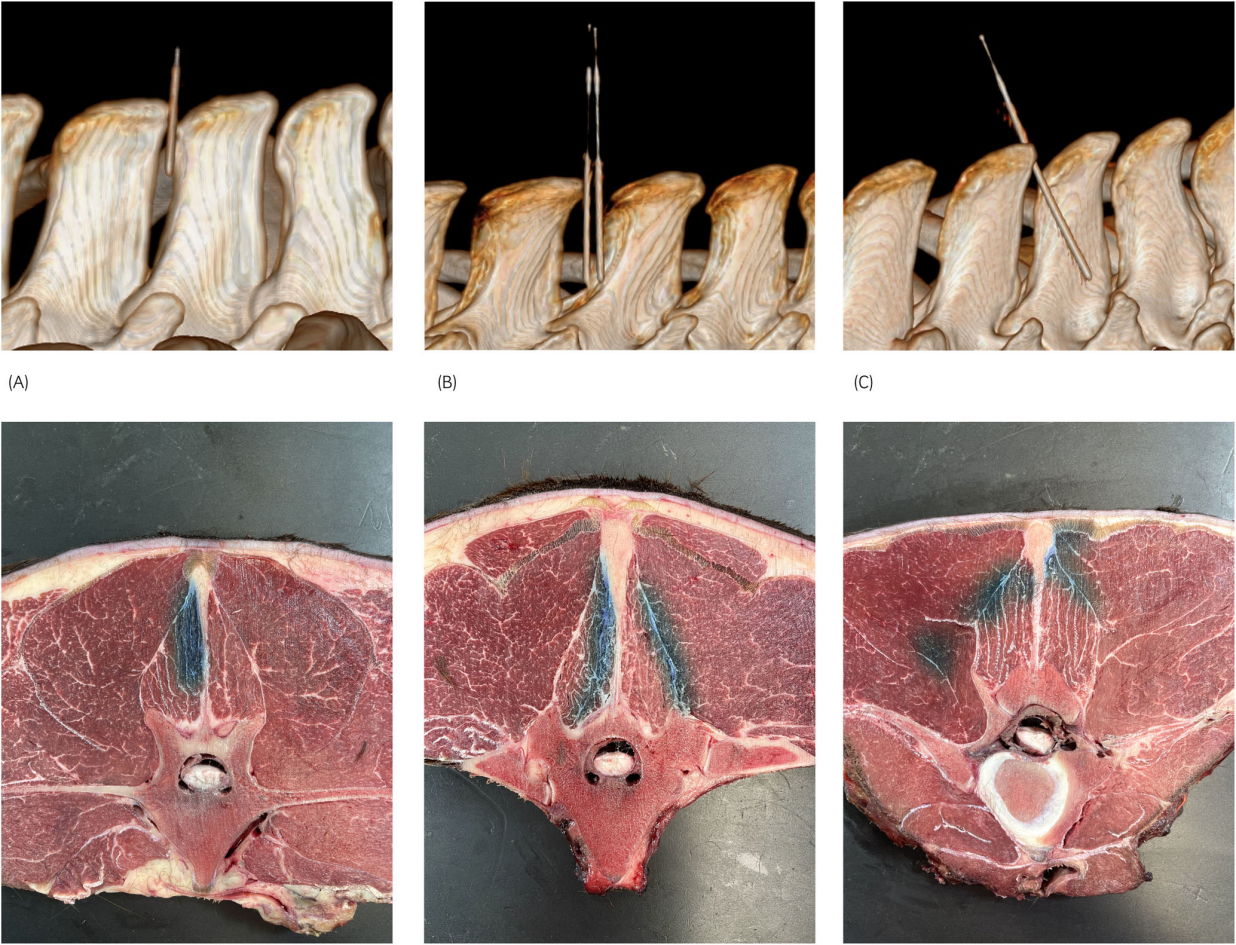
Computed tomographic illustration of three methods for local injection of the interspinal space with representative sections of the resulting methylene blue dye distribution: (A) 20G—1½″ short needle—midline approach at the level T17/T18—note blue dye in regional multifidus musculature on the left. (B) 20G—3½″ spinal needle: bilateral abaxial approach with two injections at the level T17/T18—note bilateral asymmetrical regional staining of blue dye. (C) 20G—3½″ spinal needle: unilateral oblique approach at the level L2/L3—note bilateral spread of contrast in multifidus and longissimus musculature.

Specimens were positioned on the CT couch at the height of a horse in standing posture (160 cm). Local injection was subsequently performed at four sites: interspinal space T11/12; T14/15; T17/18; L2/3. Each injection site was randomly assigned to a set combination of the selected injection technique, needle size, volume, and imaging modality to guide needle placement as illustrated in Figures 1 and 2. Each specimen was injected four times (total of 48 injections). Assessment of three injection techniques was performed (each technique 16 times), as well as two different volumes and two different needles (each needle/volume performed 24 times) (Figure 1).

#### 2.2.1 Injection technique

The following three techniques for injection of the thoracolumbar interspinal space were examined: Method 1. Midline: placing one needle in midline between two adjacent SPs; Method 2. Bilateral abaxial: placing two needles, one for left and one for right abaxial injection, ~5–10 mm abaxial to the interspinal space; Method 3. Unilateral oblique: insertion of one needle, ~10–15 mm abaxial to the interspinal space, across the interspinal space in an oblique direction from the left to the right side (Figure 2). For methods 1 and 2, the needle was oriented cranially at the level T11/12, perpendicular or slightly caudally at T14/15; T17/18 and caudally at L2/3. An injection angle of ~30°–45° to the sagittal midline was selected for the unilateral oblique technique (method 3). All injections were performed by an equine orthopaedic surgeon with 7 years of experience (DT) under the supervision of a Diplomate of the European College of Veterinary Surgeons (ECVS) and European College of Veterinary Sports Medicine and Rehabilitation (ECVSMR) (AE). The CT couch was always positioned at the same height with the right‐handed operator standing on the left side of the specimen.

#### 2.2.2 Needles for injection

Two different types of needles were used for injection: a 20G—3½″ spinal needle (acti‐fine, spine‐ject M. Schilling GmbH) or a 20G—1½″ short needle (Sterican B Braun). The needle shaft was marked in three places at equal distance along the length of the shaft using a waterproof marker pen to facilitate determination of the depth of insertion.

#### 2.2.3 Volume for injection

The volume was based on the results of a survey^7^ where equine orthopaedic specialists specified the volume they commonly used for local injection. In this survey, 5 mL was considered a small volume and 20 mL a large volume for injection. The injectate was a mixture of non‐ionic iodinated contrast 50% (Iohexol, Accupaque 300 mg/mL) and methylene blue 50% (10 mg/mL). Local injection was performed with the first 25% of the injectate deposited with the needle fully inserted. The remaining volume was continuously injected in equal amounts between the levels marked on the needle shaft as the needle was gradually withdrawn. For bilateral injection (method 2) both sides were injected with the inherent volume of 5 mL (total 10 mL) or 20 mL (total 40 mL).

#### 2.2.4 Diagnostic imaging guidance

Needles for injection were assigned to be positioned either radiographic‐guided or ultrasonographic‐guided or by palpation (no direct imaging assistance) (Figure 1) with the aid of the previously prepared clip marks.

Under direct‐radiographic guidance, the needle was inserted, and a latero‐lateral radiograph was obtained. The needle was repositioned, and further radiographs were taken as required to place the needle accurately in the interspinal space.

For injection under ultrasonographic guidance, clip‐marks were shaved to remain visible before the hair over the thoracolumbar SPs was clipped and the skin prepared for ultrasonographic evaluation. A linear transducer (7.0 MHz, MyLabFive vet, Esaote) was oriented longitudinally (in direction of the sagittal plane) over the shaved marks (T12, T16, L3). The transducer was then moved caudally, running from SP to SP until it reached the cranial aspect of the SP caudal to the interspinal space of interest. The needle was inserted cranial to the transducer at the level of the interspinal space under ultrasonographic guidance. For the unilateral oblique technique, the transducer was positioned longitudinal to midline between the SPs with the caudal aspect of the cranial SP and the cranial aspect of the caudal SP in view to facilitate visualisation of the needle as it penetrates the ISL.

The clip‐marks were also used to provide orientation for palpation of the correct interspinal spaces for injection without imaging guidance.

### 2.3 Computed tomographic analysis

Once needles were placed at the four injection sites per specimen, CT examination was performed to verify the exact needle position and correct it if required. The needle position was corrected if there was significant needle deviation to one side (method 1), the needle was positioned at the level of the DSP and not at the level of the ISL (method 2) or the needle did not cross the ISL (method 3). Computed tomographic parameters were as follows: Qalibra CT‐system equipped with a Canon Large Bore scanner whose 32‐slice detector provides 0.35 mm isotropic spatial resolution, 135 kVp, 370 mA, slice thickness 1.002 mm, slice gap 1.25 mm, field of view 40–70 cm.

Local injection with the previously prepared and drawn up injectate was performed once all needles per one specimen were positioned. For evaluation of the distribution of the injected volume, further CT scans were obtained 10, 20, and 45 min after all four injections per specimen were performed.

**FIGURE 3:**
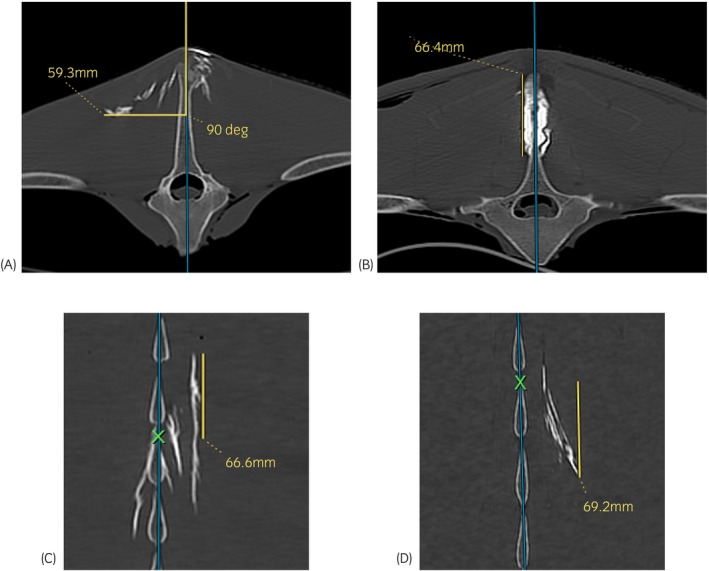
Measurement of contrast distribution based on computed tomographic imaging: (A) Lateral distribution at the level T14/T15 (method 3: Unilateral oblique injection technique, needle 20G—3½″, volume 20 mL): transverse plane image with blue reference line for orientation in the sagittal midline. The yellow line indicates the lateral extent of contrast distribution at a 90° angle to the blue reference line. (B) Dorsoventral distribution at the level T14/T15 (method 1: Injection in midline, needle 20G—1½″, volume 5 mL): transverse plane image where yellow line shows the dorsoventral spread of contrast. The yellow line is oriented parallel to the blue reference line in the sagittal plane. (C) Cranial distribution at the level T17/T18 (method 2: Bilateral abaxial injections, needle 20G—1½″, volume 20 mL): dorsal plane image with yellow line indicating cranial spread of contrast parallel to the blue reference line starting at the point of injection (green X). (D) Caudal distribution at the level T17/T18 (method 3: Unilateral oblique injection technique, needle 20G—3½″, volume 20 mL) on dorsal plane image with yellow line starting at the injection site (green X), indicating caudal spread of contrast.

CT images were later assessed in multiplanar reconstruction (image processing software Philips IntelliSpace Portal, Philips Healthcare). Contrast distribution was measured (mm) in a lateral, dorsoventral, cranial, and caudal direction to determine the maximum distance of distribution (Figure 3). The sagittal midline at the level of the widest distribution of contrast was the reference for measurement of the lateral contrast distribution. The dorsoventral contrast distribution was measured at the point of widest spread parallel to the sagittal plane. The cranial and caudal contrast distribution was determined in relation to the centre of injection (Figure 3). It was additionally documented whether contrast reached the ISL.

### 2.4 Anatomical dissection

Immediately after the last CT scan, anatomical dissection was performed to assess the distribution of the injected methylene blue dye. The skin and superficial trunk fascia were removed. The epaxial musculature (M. spinalis, M. longissimus dorsi) was transected in a transverse direction at the level of injection as well as longitudinally to be separated from the supraspinous ligament (SSL). The large epaxial muscles were reflected laterally to expose the multifidus muscles (Mm. multifidi). The multifidus fascicles were carefully dissected and reflected off the SPs and ISLs. A tape measure was used to determine the lateral, dorsoventral, cranial, and caudal extent of muscle staining in millimetres (mm) starting from the point of injection. Based on gross visualisation, staining of the abaxial ISL layers and the ISL adipose core was assessed and documented.^16^ With the aid of the tape measure (mm), dorsoventral staining of the ISL layers was categorised as not stained, partially stained with less than 50% of the ISL stained, partially stained with more than 50% of the ISL stained, and ISL completely stained. Staining of the adipose core was further categorised as not stained or stained. Additionally, staining of the SSL, the SPs, and the thoracolumbar articular process joint capsule was recorded. The SSL was considered as stained where ≥1 cm of the SSL was stained, as measured from the point of injection in a cranial and/or caudal direction. SPs were categorised as stained if the periosteal surface showed evidence of blue dye, and the thoracolumbar articular process joints where there was methylene blue staining of the joint capsule.

### 2.5 Data analysis

Data were recorded in Microsoft Excel (Microsoft Excel 2022, Microsoft Corporation) and analysed using SPSS (IBM SPSS Statistics 26). Injection sites where contrast or methylene blue could not clearly be referred to a specific injection or were mixed up between adjacent sites were excluded from further data analysis, and the injection was repeated in a new specimen. Descriptive analysis was performed for left/right lateral, dorsoventral, cranial, and caudal CT‐contrast distribution after 10, 20, and 45 min depending on the method (approach) used for injection as well as the needle, volume, and imaging guidance (four groups). Data were visually inspected for normal distribution. Since CT data were not normally distributed, the Mann–Whitney *U* test was used to compare two groups and the Kruskal–Wallis test for more than two investigated categories.

**FIGURE 4:**
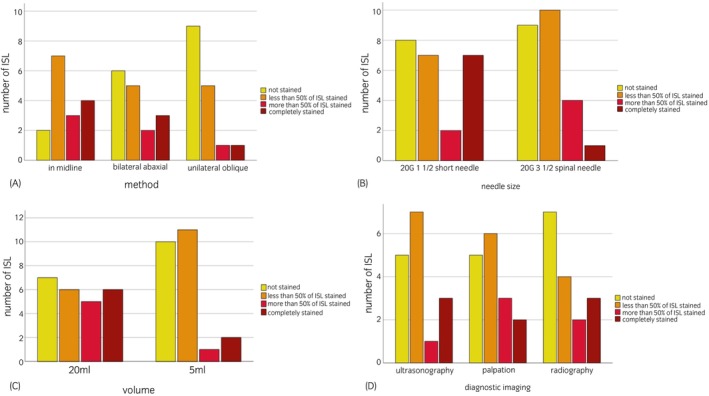
Bar chart illustrating dorsoventral methylene blue staining of the interspinous ligament (ISL) depending on the method (A), needle size (B), volume (C) diagnostic imaging guidance, and palpation (D) used for local injection (48 injection sites).

Descriptive analysis was also performed for the assessment of the methylene blue dye distribution. Chi‐squared tests or exact Fisher's tests (if at least 25% of cells had five or less expected observations) were applied to examine the association between the method (in‐midline, bilateral abaxial, unilateral oblique), as well as the choice of needle, volume and imaging guidance used for injection and the staining (variables: complete staining, partial staining >50%, partial staining <50%, no staining) of the local anatomical structures, represented in Figure 4. The *p*‐values <0.05 were considered significant. If more than two categories were present and all categories were compared with each other the Dunnett's post hoc test was applied.

## 3 RESULTS

### 3.1 Computed tomographic measurements

Two specimens were excluded as severe SP pathology was identified during the initial radiographic examination. In the initial CT examination, the needle position was found to be aberrant in four injection sites (needle inserted under radiographic guidance *n* = 2, ultrasonographic guidance *n* = 1 and palpation *n* = 1). The needle was subsequently repositioned using the previously assigned method and imaging guidance. Injection sites where the boundaries of contrast or methylene blue dye staining could not be evaluated due to overlap between adjacent injection sites (*n* = 16) were excluded from further data analysis and were repeated. A total of four additional spinal specimens were prepared as described for this purpose. As no overlap of contrast or methylene blue dye staining was observed in the repeated injections, data was included for further analysis.

#### 3.1.1 Injection technique

**FIGURE 5:**
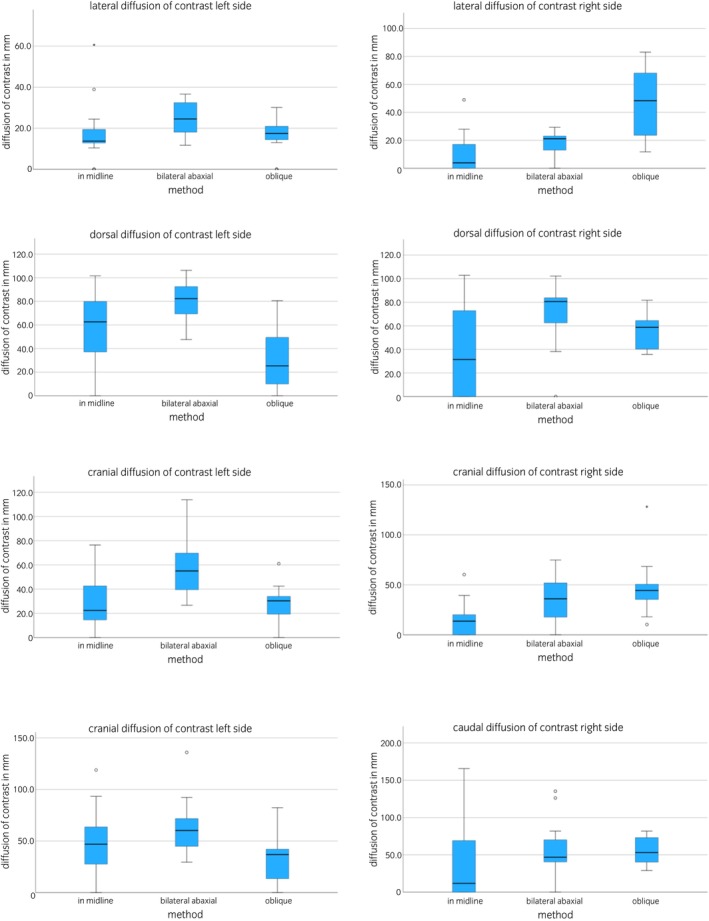
Box plots illustrating computed tomographic contrast distribution (48 injection sites) in equine spine specimens depending on the method used for local injection. Black lines represent the median. Whiskers represent values outside the interquartile range. Outliers are indicated by asterisks (*).

Contrast distribution over time was documented 10, 20, and 45 min after injection (Tables 1, 2, 3). Lateral, dorsoventral, cranial, and caudal contrast distribution was further assessed in relation to the method used for local injection. The distribution of contrast varied depending on the method used for injection, with different patterns of contrast distribution observed between the left and the right side of the vertebral column (Table 1; Figure 5). There was significantly less lateral diffusion on the left side of the interspinal space with method 1 (midline) when compared with method 2 (bilateral abaxial), with no significant difference concerning lateral contrast distribution on the right side (Table 1; Figure 5). Lateral contrast distribution was limited to the multifidus musculature when method 1 was used. Additionally, there was significantly less dorsoventral contrast distribution following method 1 when compared with method 2 on the right side. Less cranial diffusion was observed bilaterally with method 1, but there was no significant difference regarding contrast distribution between method 1 and 2 in a caudal direction (Table 1; Figure 5).

**TABLE 1:** Median CT contrast distribution in a lateral, dorsoventral, cranial, and caudal direction, 45 min after local injection of the interspinal space (48 injection sites) using three different methods for injection.

Spread of contrast after injection	Method 1 midline, *n* = 16	Method 2 bilateral abaxial, *n* = 16	Method 3 unilateral oblique, *n* = 16	Time (min)
Lateral left side median mm (range mm)	12.2 (8.5–15.6)	21.2 (14.2–29.6)	16.2 (11.5–18.5)	10
	12.4 (10.4–16.3)	22.2 (14.4–32.9)	16.4 (11.5–19.8)	20
	13.7^a^ (12.8–19.4)	24.5^a^ (16.1–32.6)	17.4 (13.0–21.0)	45
Lateral right side median mm (range mm)	0.8 (0.0–11.4)	17.0 (11.7–20.0)	46.2 (16.5–66.3)	10
	0.9 (0.0–16.4)	18.4 (12.5–21.6)	46.5 (17.0–67.7)	20
	3.9^b^ (0.0–11.7)	21.1 (12.9–23.8)	48.3^b^ (21.0–68.1)	45
Dorsoventral left side median mm (range mm)	58.5 (34.1–71.1)	75.2 (60.6–86.4)	20.6 (53.0–44.7)	10
	61.8 (36.4–74.6)	81.1 (66.5–88.7)	25.3 (5.3–51.1)	20
	62.6 (37.2–79.9)	82.3^c^ (68.3–93.4)	25.3^c^ (5.9–51.7)	45
Dorsoventral right side median mm (range mm)	19.7 (0.0–64.6)	71.1 (58.2–80.0)	41.1 (34.1–54.6)	10
	23.8 (0.0–69.3)	71.1 (61.1–80.6)	54.7 (36.5–64.1)	20
	31.5^d^ (0.0–72.9)	80.5^d^ (61.7–84.0)	58.7 (38.8–64.6)	45
Cranial left side median mm (range mm)	15.9 (9.5–36.4)	47.3 (30.8–54.6)	25.7 (14.2–29.6)	10
	19.2 (13.0–40.4)	47.7 (34.6–56.4)	26.8 (13.6–32.7)	20
	22.4^e^ (14.7–42.7)	55.0^e;f^ (35.8–72.9)	30.3^f^ (14.8–34.0)	45
Cranial right side median mm (range mm)	9.6 (0.0–19.0)	32.3 (12.2–43.2)	37.4 (26.3–44.5)	10
	11.5 (0.0–20.0)	32.4 (14.3–52.0)	42.1 (31.2–51.7)	20
	13.7^g;h^ (0.0–20.1)	36.0^g^ (17.5–52.4)	44.3^h^ (35.3–52.1)	45
Caudal left side median mm (range mm)	42.7 (20.4–60.4)	49.3 (31.5–63.3)	28.5 (11.5–35.5)	10
	44.6 (26.1–61.9)	54.3 (32.0–64.6)	28.8 (12.3–36.7)	20
	47.0 (27.6–63.6)	60.2^i^ (40.3–72.4)	36.8^i^ (13.4–43.2)	45
Caudal right side median mm (range mm)	8.90 (0.0–57.0)	42.6 (29.5–55.1)	46.8 (36.1–67.1)	10
	9.0 (0.0–57.4)	44.5 (34.9–66.5)	51.0 (37.4–68.5)	20
	11.8 (0.0–69.0)	46.8 (40.5–73.3)	53.0 (38.4–73.3)	45

*Note*: Significant differences are marked with superscript letters. Superscript letters indicate statistically significant *p*‐values <0.05: a = 0.02; b = 0.001; c = 0.001; d = 0.01; e = 0.003; f = 0.005; g = 0.01; h = 0.001; i = 0.02.

**TABLE 2:** CT contrast distribution in a lateral, dorsoventral, cranial and caudal direction, 45 min after local injection of the interspinal space (48 injection sites) using two different needles.

Spread of contrast after injection	20G—1½″ short needle, *n* = 24	20G–3½″ spinal needle, *n* = 24	Time (min)
Lateral left side median mm (range mm)	16.0 (10.9–22.4)	14.7 (9.6–23.8)	10
	16.3 (11.9–22.9)	16.4 (12.0–24.3)	20
	18.1 (13.7–24.5)	17.7 (12.3–25.8)	45
Lateral right side median mm (range mm)	16.3 (10.3–22.1)	15.2 (0.0–57.7)	10
	16.9 (12.2–24.3)	16.7 (0.0–58.7)	20
	19.5 (13.0–26.8)	17.3 (3.9–59.2)	45
Dorsoventral left side median mm (range mm)	51.7 (30.0–72.3)	59.7 (16.2–75.5)	10
	60.0 (36.7–74.2)	61.2 (16.7–79.9)	20
	62.0 (38.9–81.4)	65.2 (17.9–81.2)	45
Dorsoventral right side median mm (range mm)	44.7 (26.5–64.1)	49.6 (15.9–79.1)	10
	54.7 (35.8–66.7)	60.0 (16.8–79.6)	20
	60.6 (38.5–73.2)	61.1 (17.7–82.0)	45
Cranial left side median mm (range mm)	27.8 (15.9–38.4)	27.9 (9.1–49.6)	10
	29.3 (22.0–41.8)	31.9 (11.9–53.7)	20
	32.3 (25.3–43.3)	33.1 (14.8–55.9)	45
Cranial right side median mm (range mm)	24.6 (10.9–43.2)	22.1 (0.0–36.8)	10
	29.4 (15.3–43.8)	24.7 (4.0–45.2)	20
	33.8 (16.6–47.1)	26.3 (5.2–46.5)	45
Caudal left side median mm (range mm)	42.0 (28.9–65.6)	34.5 (16.0–51.5)	10
	44.6 (29.7–68.2)	36.8 (18.9–55.4)	20
	49.1 (35.3–79.9)	42.7 (20.5–62.0)	45
Caudal right side median mm (range mm)	38.6 (23.4–48.3)	43.3 (0.0–64.3)	10
	39.2 (26.4–51.1)	48.9 (0.0–67.4)	20
	40.6 (28.7–55.3)	52.8 (0.0–73.1)	45

**TABLE 3:** CT contrast distribution in a lateral, dorsoventral, cranial, and caudal direction, 45 min after local injection of the interspinal space (48 injection sites) using two different volumes for injection.

Spread of contrast after injection	Small volume 5 mL, *n* = 24	Large volume 20 mL, *n* = 24	Time (min)
Lateral left side median mm (range mm)	16.2 (10.1–20.2)	14.7 (10.5–24.4)	10
	16.9 (12.0–21.0)	15.7 (11.9–25.9)	20
	18.0 (13.7–22.2)	17.1 (13.0–27.8)	45
Lateral right side median mm (range mm)	13.1 (0.0–23.8)	17.7 (11.6–26.5)	10
	15.0 (0.0–25.5)	17.7 (12.3–27.6)	20
	16.8 (0.0–27.5)	20.3 (13.4–28.6)	45
Dorsoventral left side median mm (range mm)	50.8 (26.8–64.2)	65.6 (24.4–79.4)	10
	58.5 (30.9–69.4)	69.1 (30.3–81.7)	20
	60.0 (31.3–72.6)	74.1 (32.4–87.6)	45
Dorsoventral right side median mm (range mm)	34.1 (0.0–49.6)	64.1 (43.2–77.9)	10
	36.8 (0.1–50.8)	66.7 (55.6–80.6)	20
	38.5^a^ (0.1–55.8)	72.0^a^ (61.2–83.7)	45
Cranial left side median mm (range mm)	25.8 (14.8–32.8)	33.8 (14.6–51.0)	10
	26.8 (17.0–40.0)	36.7 (19.5–53.7)	20
	30.6 (17.5–42.4)	38.3 (22.1–59.5)	45
Cranial right side median mm (range mm)	14.8 (0.0–41.2)	30.2 (13.6–41.0)	10
	17.1 (0.0–42.7)	32.0 (16.4–52.5)	20
	17.8 (0.0–44.6)	35.7 (17.3–55.2)	45
Caudal left side median mm (range mm)	34.3 (20.8–47.7)	45.5 (28.9–65.6)	10
	35.3 (25.8–51.8)	46.4 (29.7–71.3)	20
	40.6^b^ (28.6–57.5)	54.7^b^ (35.7–73.7)	45
Caudal right side median mm (range mm)	29.6 (0.0–42.2)	53.5 (38.1–67.8)	10
	33.4 (0.0–46.6)	57.0 (39.2–74.2)	20
	35.2^c^ (0.0–47.8)	67.9^c^ (42.6–77.2)	45

*Note*: Significant differences are marked with superscript letters. Superscript letters indicate statistically significant *p*‐values <0.05: a = 0.001; b = 0.005; c = 0.001.

Comparing method 1 with method 3 (unilateral oblique) there was significantly more lateral spread of contrast with method 3 than with method 1 on the right side. Contrast diffusion extending beyond the level of the multifidus musculature into the M. longissimus was observed on the right side using method 3. Cranial contrast diffusion on the right was significantly larger for method 3 than for method 1, with no significant differences identified for the dorsoventral or caudal spread of contrast (Table 1; Figure 5).

The comparative analysis of method 2 and 3 did not identify significant differences in lateral contrast distribution. There was, however, significantly more dorsoventral contrast following injection using method 2 compared with method 3 on the left. The cranial distribution of contrast was significantly different between method 2 and 3, with further spread of contrast following injection with method 2 on the left side. The caudal spread of contrast on the left side was also more extensive with method 2 when compared with method 3, with no significant difference detected for the right side (Table 1; Figure 5).

#### 3.1.2 Needles for injection

Considering the type of needle used for injection, there was no significant difference in CT contrast diffusion between the 20G—3½″ spinal needle and the 20G—1½″ short needle (Table 2).

#### 3.1.3 Volume for injection

Whilst there was no significant difference in the lateral spread of contrast with different volumes injected, there was significantly more dorsoventral contrast diffusion on the right side with a volume of 20 mL when compared with a volume of 5 mL. The larger volume of 20 mL did not result in significantly wider contrast distribution in a cranial direction. There was, however, significantly more contrast in a caudal direction on both sides when a larger volume (20 mL) was injected (Table 3).

### 3.2 Anatomical dissection

#### 3.2.1 Interspinous ligament

During anatomical dissection, methylene blue staining of the different layers (Figure 4) as well as the adipose core of the ISL was evaluated.

The adipose core of the ISL was most likely dyed with method 1 (37.5%; *n* = 6 of 16; *p* = 0.01). Method 2 had the most even and the most extended distribution of the injected volume on both sides of the interspinous space, but with an abaxial pattern and significantly less staining of the adipose core of the ISL in 18.8% (*n* = 3 of 16; *p* = 0.18). Method 3 resulted in staining of the ISL core in 6.3% (*n* = 1 of 16; *p* = 0.008) of injections.

Concerning the length of the needle, staining of the ISL adipose core was achieved in 33.3% (*n* = 8 of 24) of cases with the 20G—1½″ short needle and 8.3% (*n* = 2 of 24) of cases with the 20G—3½″ spinal needle (*p* = 0.03). The larger volume of 20 mL was more likely to stain the ISL adipose core (29.2%, *n* = 7 of 24) when compared with a volume of 5 mL (12.5%; *n* = 3 of 24; *p* = 0.03). Staining of more than one ISL with the ISL cranial to the injection site methylene blue dyed was observed in two injections where the injection was performed using method 2, with a volume of 20 mL (20G—1½″: *n* = 1; 20G—3½″: n = 1; both inserted under ultrasonographic guidance).

#### 3.2.2 Supraspinous ligament and spinous processes

Staining of the SSL was most likely achieved using method 3 with 10 mm of the SSL cranial and caudal to the injection site dyed in 43.8% of cases (*n* = 7 of 16; *p* = 0.03). The small volume of 5 mL stained the SSL and the area 10 mm cranial to the injection site in 41.7% (*n* = 10 of 24) and the large volume of 20 mL in 20.8% of cases (*n* = 5 of 24; *p* = 0.2). Ultrasonographic needle guidance resulted in the most extended SSL staining (10 mm cranial, 56.3%, *n* = 9 of 16; 10 mm caudal, 43.8%, *n* = 7 of 16; *p* = 0.03).

The osseous surface of the SP summit was dyed on both sides in 50.0% of cases (*n* = 8 of 16) using method 1 and in 37.5% (*n* = 6 of 16) with method 2, whereas method 3 was less likely to stain the SP (6.3%; *n* = 1 of 16; *p* = 0.001). SP staining was additionally achieved using a 20G—1½″ short needle in 41.7% of injections (*n* = 10 of 24) and using a smaller volume of 5 mL in 33.3% (*n* = 8 of 24; *p* = 0.5).

#### 3.2.3 Thoracolumbar articular process joints

Methylene blue staining of the thoracolumbar articular process joint capsule was bilaterally identified in 66.7% (*n* = 16 of 24) injection sites when the 20G—3½″ spinal needle was used and in 41.7% (*n* = 10 of 24; *p* = 0.07) sites when the 20G—1½″ short needle was used. The thoracolumbar articular process joints of the spinal segment cranial to the injection site were bilaterally dyed more often when the 20G—3½″ spinal needle was inserted (25.0%; *n* = 6 of 24) instead of the 20G—1½″ short needle (4.2%; *n* = 1 of 24; *p* = 0.04). Similarly, the adjacent thoracolumbar articular process joints caudal to the injection site were stained with the 20G—3½″ spinal needle (bilateral: 41.6%; *n* = 10 of 24) significantly more often when compared with the 20G—1½″ short needle (unilateral: 12.6%; *n* = 3 of 24; *p* = 0.005). Two thoracolumbar articular process joints caudal to the injection site were bilaterally methylene blue dyed in 20.8% (*n* = 5 of 24) of injections when the 20G—3½″ spinal needle was used (*p* = 0.005).

Considering the volume used for injection, the thoracolumbar articular process joint cranial to the injection site was stained in 20.8% (unilateral 20.8%; *n* = 5 of 24) with 5 mL and 16.7% (bilateral: *n* = 4 of 24) when a volume of 20 mL was injected (*p* = 0.5). The two thoracolumbar articular process joints caudal to the injection site were stained in 29.2% (bilateral: *n* = 7 of 24) of cases when a volume of 20 mL was used and in 16.6% (bilateral: *n* = 4 of 24; *p* = 0.5) with injection of the 5 mL volume. Diffusion ventral to the level of the thoracolumbar articular process joints was not observed.

#### 3.2.4 Diagnostic imaging guidance

With the ISL determined as the main target for injection, the radiographic needle guidance as well as the ultrasonographic guidance led to complete staining of the ISL including the adipose core in 18.8% (*n* = 3 of 16) of injection sites with 12.5% (*n* = 2 of 16; *p* = 0.5) recorded for palpation alone (Figure 4D).

## 4 DISCUSSION

The evaluation of different techniques for the local injection of the equine thoracolumbar interspinal spaces without severe SP pathology identified that the ISL was most likely to be injected when a 20G—1½″ needle was placed in between the SPs in midline (method 1) and a volume of 5 mL was injected. Based on the results of this study, radiographic needle guidance is not required as ultrasonographic guidance or palpation is sufficient for needle positioning at the level of the interspinal space.

Whilst the successful application of an injectate exactly in midline (method 1) has been described to be impractical and the adjacent epaxial musculature is likely to be injected, it remains the method with the highest likelihood of complete ISL injection (25%) based on the current study.^6^ Method 2 (bilateral abaxial) facilitates the most even and the most extended distribution of the injected contrast volume on both sides of the interspinal space (Table 1; Figure 5), but with an abaxial pattern and a significantly lower chance of reaching the adipose core of the ISL, where most sensory innervation has been identified.^16^, ^17^ Passing the needle in an oblique direction (unilateral oblique) across the interspinal space (method 3) is used by equine orthopaedic specialists (personal communication^7^) for the following reasons: (1) It is easier to insert the needle between the SPs particularly in cases where the midline approach (method 1) is hampered by severe reduction of the interspinal space width with impingement of SPs. (2) As the needle is withdrawn across the interspinal space and the volume is injected simultaneously a high likelihood of ISL injection is expected.^7^ However, based on the results of the current study, method 3 resulted in complete staining of all ISL layers in only 6.3% of cases and an asymmetrical distribution of the injected volume was identified with most of the injectate localised in the epaxial musculature (Mm. multifidi, M. longissimus) on the contralateral (right) side of the vertebral column. Needle insertion in a steeper angle as well as bilateral oblique needle insertion might improve results.

Asymmetrical, abaxial distribution of the injectate to one side was also evident in 88% (*n* = 14 of 16) of cases where method 1 was used (Figure 2A). Possible explanations could be the position of the person performing the injection (standing on the left side of the horse) as well as the degree of narrowing of the interspinal space causing mild deviation of the needle to one side.^6^ The abaxial contrast distribution observed with method 1 was, however, limited to the regional multifidus muscles whereas method 2 (bilaterally) and method 3 (unilaterally on the right) led to extensive distribution of the injected volume along the multifidus muscle fascicles and the thoracolumbar fascia in a lateral, cranial and caudal direction with a lower likelihood of simultaneous ISL injection (Figure 2B,C). Based on this result, methods 2 and 3 appear less accurate for the local injection of the equine thoracolumbar interspinal space. Both methods may, however, be considered for local treatment in certain cases, depending on whether the dorsal, middle or ventral part of the SP is most affected by pathological changes. Additionally, pre‐operative injection of the regional musculature (method 2) and the SSL (method 3) with local anaesthetic agents appears feasible. Both methods may result in a wider distribution of the local anaesthetic reaching the ISL, SSL, SP and the epaxial musculature as desired for pre‐operative analgesia.

The thoracolumbar articular process joints were stained more frequently when the longer 20G—3½″ spinal needle was used. Whilst impingement of the SP and osteoarthritis of the thoracolumbar articular process joints often occur simultaneously, a 20G—1½″ short needle should be selected if the aim is to inject the interspinous ligament in the interspinal space, particularly for diagnostic purposes in horses with ORSPs.

Concerning the volume used for injection, there was further distribution of the injectate, particularly in a caudal direction, when the larger volume of 20 mL was used. As described for the erector spinae plane block, the use of a longer needle and a larger volume can cause extended distribution of the injectate along fascial planes, for example, between the fascial layers of the multifidus muscles or the transverse processes and the erector spinae muscle.^9^ Whilst this wide volume distribution is beneficial for perioperative local analgesia, it is not specific for diagnostic purposes and a smaller volume of 5 mL should be selected for local injection of the interspinal space.^18^

Diagnostic imaging is frequently utilised to diagnose impingement of SPs as well as to aid correct needle positioning for thoracolumbar injections, with radiographic examination being the modality most commonly employed.^7^, ^19^ The results of the current study confirm that the repeated radiographic examination for assessment of the correct needle placement in addition to the previously performed diagnostic radiographic procedure bears no advantage. The use of ultrasonography and palpation (68.8% of ISL partially or completely stained) was sufficient and resulted in an accurate needle position more frequently when compared with the direct radiographic control (56.3% of ISL partially or completely stained). Inaccuracy of the radiographically guided needle position is most likely related to the two‐dimensional quality of the latero‐lateral projection with the potential for inadvertent variation of the x‐ray beam angle and geometric distortion of the interspinal space.^20^ Based on the anatomical orientation of the SPs, the ultrasound‐guided needle insertion may be further refined with the transducer positioned over the caudal aspect of the SP cranial to the interspinal space to be injected and the needle inserted caudal to the transducer in the mid‐thoracic spine. It can additionally be assumed that the level of experience and technical skill of the individual operator has an impact on the accuracy of the ultrasound‐guided injection.

Where narrowing of the interspinal space occurs in line with the so‐called Baastrup Syndrome in humans, local injections can be performed under fluoroscopic guidance.^21^, ^22^ This imaging modality is, however, not feasible for thoracolumbar injections in the area of the equine thoracolumbar spine. As the direct radiographic control was the least accurate method for guided needle insertion but obtaining thoracolumbar radiographs involves considerable radiation exposures, the method should be discouraged based on the principles of radiation safety (ALARA principle). Alternatively, insertion of the needle can be performed with higher accuracy using the described techniques for ultrasonographic guidance or palpation.^23^

Limitations include the absence of severe SP pathology as well as the ex vivo nature of the study and the limited number of interspinal spaces injected for each evaluated method. The choice of a water‐soluble contrast agent (iohexol) and methylene blue dye solution for the assessment of local injection has been previously described.^14^, ^24^ Most likely, a wider distance between injection sites would have prevented overlap of the injected volume and avoided repeated injection. The authors suspect that the diffusion pattern might differ in the in vivo situation as the distribution of the injected volume is influenced by the muscular haemodynamics and the sub‐atmospheric pressure between the fascial planes in vivo.^13^, ^14^

In conclusion, the evaluation of different techniques for the local injection of the thoracolumbar interspinal space in horses without severe SP changes confirmed that the midline approach remains the most accurate technique when performed with a 20G—1½″ needle and a volume of 5 mL. Radiographic assessment has not proven to be reasonable for imaging‐guided thoracolumbar injections in this study, but ultrasonography or palpation should be considered. In contrast to the authors' hypothesis, the unilateral oblique needle insertion across the interspinal space did not sufficiently reach the well‐innervated aspects of the ISL. Whether this technique is obsolete or requires adjustment of the exact point of needle insertion and angulation warrants further investigation. Additionally, investigation of the described techniques for local injection in SPs showing more severe pathological changes is warranted.

## FUNDING INFORMATION

No external funding was received.

## CONFLICT OF INTEREST STATEMENT

None of the authors has any financial or personal relationships that could inappropriately influence or bias the content of the article.

## AUTHOR CONTRIBUTIONS

**Dorothea Tress:** Writing – original draft; conceptualization; investigation; data curation; software; visualization. **Simon Hennessy:** Writing – review and editing. **Roswitha Merle:** Data curation; formal analysis; software. **Katharina Charlotte Jensen:** Data curation; formal analysis; software. **Christoph Lischer:** Supervision. **Anna Ehrle:** Conceptualization; writing – original draft; supervision; project administration; methodology; validation.

## DATA INTEGRITY STATEMENT

Dorothea Tress had full access to all data in the study and takes responsibility for the integrity of the data and the accuracy of the data analysis.

## ETHICAL ANIMAL RESEARCH

Ethical approval was obtained (LAGeSo Berlin 004/19).

## INFORMED CONSENT

Owners gave consent for their animals' inclusion in the study.

## Data Availability

The data that support the findings of this study are openly available in figshare (https://figshare.com/articles/dataset/SPSS_section/28927346 and https://figshare.com/articles/dataset/SPSS_CT/28927358).
